# It's the fiber, not the fat: significant effects of dietary challenge on the gut microbiome

**DOI:** 10.1186/s40168-020-0791-6

**Published:** 2020-02-11

**Authors:** Kathleen E. Morrison, Eldin Jašarević, Christopher D. Howard, Tracy L. Bale

**Affiliations:** 1 Center for Epigenetic Research in Child Health and Brain Development, Department of Pharmacology, University of Maryland School of Medicine, HSF3, room 9-171, 670 W. Baltimore St., Baltimore, MD 21201 USA; 2 Center for Epigenetic Research in Child Health and Brain Development, Department of Psychiatry, University of Maryland School of Medicine, HSF3, room 9-171, 670 W. Baltimore St., Baltimore, MD 21201 USA

**Keywords:** Microbiome, Metabolism, Diet, Aging, Sex differences

## Abstract

**Background:**

Dietary effects on the gut microbiome play key roles in the pathophysiology of inflammatory disorders, metabolic syndrome, obesity, and behavioral dysregulation. Often overlooked in such studies is the consideration that experimental diets vary significantly in the proportion and source of their dietary fiber. Commonly, treatment comparisons are made between animals fed a purchased refined diet that lacks soluble fiber and animals fed a standard vivarium-provided chow diet that contains a rich source of soluble fiber. Despite the well-established critical role of soluble fiber as the source of short chain fatty acid production via the gut microbiome, the extent to which measured outcomes are driven by differences in dietary fiber is unclear. Further, the interaction between sex and age in response to dietary transition is likely important and should also be considered.

**Results:**

We compared the impact of transitioning young adult and 1-year aged male and female mice from their standard chow diet to a refined low soluble fiber diet on gut microbiota community composition. Then, to determine the contribution of dietary fat, we also examined the impact of transitioning a subset of animals from refined low-fat to refined high-fat diet. We used a serial sampling strategy coupled with 16S rRNA marker gene sequencing to examine consequences of recurrent dietary switching on gut microbiota community dynamics. Analysis revealed that the transition from a chow diet to a refined diet that lacks soluble fiber accounted for most of the variance in community structure, diversity, and composition across all groups. This dietary transition was characterized by a loss of taxa within the phylum Bacteroidetes and expansion of Clostridia and Proteobacteria in a sex- and age-specific manner. Most notably, no changes to gut microbiota community structure and composition were observed between mice consuming either refined low- or high-fat diet, suggesting that transition to the refined diet that lacks soluble fiber is the primary driver of gut microbiota alterations, with limited additional impact of dietary fat on gut microbiota.

**Conclusion:**

Collectively, our results show that the choice of control diet has a significant impact on outcomes and interpretation related to diet effects on gut microbiota. As the reduction of soluble fiber may influence synthesis of microbial metabolites that are important for regulating metabolic, immune, behavioral, and neurobiological outcomes, additional studies are now needed to fully delineate the contribution of fat and fiber on the gut microbiome.

**Video Abtract.**

## Background

The increased availability and consumption of energy-dense foods is a key contributor to the global obesity epidemic [1]. The most common rodent model of diet-induced obesity is the consumption of diets that contain 45–60% of energy from dietary fat. This dietary intervention recapitulates key features of metabolic syndrome observed in humans, including body weight gain, increased adiposity, insulin resistance, hyperglycemia, hypertension, and dyslipidemia [2–8]. The gut microbiome has become recognized as a critical intermediary between diet and the metabolic and immune mechanisms contributing to obesity [2, 9–13]. One proposed mechanism linking the gut microbiota to obesity involves high-fat diet enrichment of gut microbial communities that exhibit an increased capacity for energy harvest and storage [2, 10, 11, 14, 15]. However, the data supporting significant diet and gut microbiota interactions are largely based on comparisons between animals fed diets that are not comparable based on nutritional composition [7]. Specifically, high-fat diets are formulated as a refined diet in which the source and proportion of every purified ingredient is known and controlled, and outcomes in high-fat-diet-fed animals are most commonly compared to animals fed an unrefined chow diet [2–4, 11, 16]. Chow diet is a catch-all descriptor for any in-house, institutionally provided vivarium diet. Unlike the refined diets, there is no standardization among chow diets, as nutritional composition is dictated by the market cost of individual ingredients, resulting in variability across batches, lots, and manufacturers [9, 10]. Among the various differences between chow and refined diets, the source of dietary fiber is the most important with respect to outcomes on the gut microbiome and metabolism [5]. Dietary fibers are broadly classified as soluble or insoluble, with different types in each category [5]. Microbiota ferment soluble fibers to produce short chain fatty acids (SCFAs). In turn, SCFAs provide a major source of energy for colonocytes, promote growth of commensal microbiota and contain outgrowth of pathogenic bacteria, decrease adipose storage, improve insulin sensitivity, and decrease local and systemic inflammation [5]. Conversely, insoluble fibers are poorly fermented and therefore do not provide any of the abovementioned benefits [6]. Given that chow diets provide both soluble and insoluble fiber while the most commonly used refined diets contain only the insoluble fiber cellulose, the disparity between sources of fiber may have important experimental consequences given their well-established effects on the gut microbiota and metabolism [6–8, 17].

Moreover, understanding how these dietary components interact with the microbiome has a heightened sense of importance in aging, as the diet of elderly individuals is more likely to be low in soluble fiber [18]. Despite evidence showing that individual differences in response to dietary challenges are driven by age and sex, existing studies have primarily focused on outcomes in adult males [19–21]. Aging-related alterations in the intestinal microbiota are associated with systemic inflammation in elderly populations, and modulating microbiota community composition and function through dietary intervention has been suggested as a therapeutic method to promote or restore health among aging populations [18, 22]. Studies on sex differences in the regulation of metabolism have shown that pre-menopausal women show higher protection against high-fat-diet-induced obesity relative to men and post-menopausal women [20]. These differences in the susceptibility to obesity and metabolic syndrome are associated with sex differences in circulating gonadal hormones, immunity, and metabolism [18, 20, 22]. Even in studies that carefully examined age- and sex-specific effects of high-fat diet consumption on metabolism and gut microbiota, comparisons were still between mice fed a refined high-fat diet and those fed a chow diet [23, 24]. Given the compositional differences between unrefined chow and refined diets, it is not clear whether these outcomes are driven by dietary fat or other dietary components such as fiber [7, 23, 24].

**Fig. 1:**
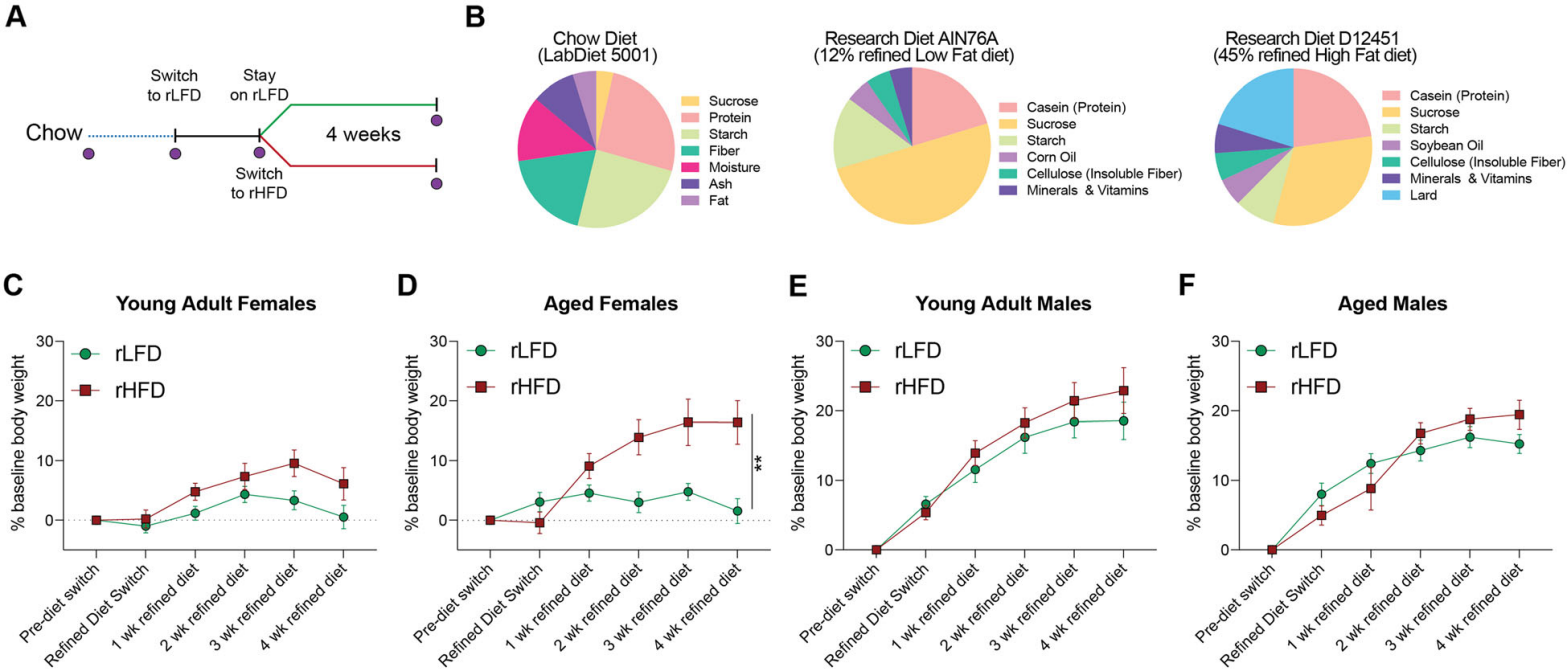
Lack of soluble fiber and increased fat in diet formulations influence weight gain in mice in an age- and sex-specific manner. **a** Schematic of the experimental study design. Young adult (17 weeks old) and 1-year aged (60 weeks old) C57Bl/6:I129 males and females consuming a chow diet were switched to a refined low-fat diet (rLFD) for 1 week to acclimate. Following acclimation to a refined diet, half of the animals remained on rLFD while the other half was switched onto a 45% refined high-fat diet (rHFD). Purple circles denote times when fecal samples were collected. Animals were co-housed and therefore all analysis is conducted at the level of the cage to control for co-housing effects (*N* = 3 cages/age/sex/diet, total *N* = 92 mice). **b** Composition of diet nutritional composition and ingredients for the chow, rLFD, and rHFD, demonstrating differences in fiber source and quantity between chow and refined diets. **c**–**f** To determine the impact of dietary switching in young adult and aged males and females, weekly body weights were collected prior to refined diet switch, 1 week following switch to rLFD, and weekly measurements during consumption rLFD or rHFD. **c** Body weight was significantly changed over time in young adult females (RM ANOVA, main effect of time, *F*_5, 110_ = 15.39, *P* < 0.000, main effect of diet, *F*_1, 22_ = 2.920, *P* = 0.1016, *N* = 24, time × diet interaction, *F*_5, 110_ = 2.782, *P* = 0.021). **d** Body weight of aged females was significantly changed over time (RM ANOVA, main effect of time, *F*_5, 90_ = 17.43, *P* < 0.0001, *N* = 20), across diets (RM ANOVA, main effect of diet, *F*_1, 18_ = 6.800, *P* = 0.0178, *N* = 20), and their interaction (RM ANOVA, time × diet, *F*_5, 90_ = 12.02, *P* = < 0.0001, *N* = 20). Post hoc analysis revealed aged females fed rHFD weighed more at 2 (*t*_108_ = 3.499, *P* = 0.0041), 3 (*t*_108_ = 3.748, *P* = 0.0017), and 4 (*t*_108_ = 4.781, *P* < 0.0001) weeks compared with rLFD-fed aged females. **e** Body weight was significantly changed over time in young adult males (RM ANOVA, main effect of time, *F*_5, 105_ = 88.146, *P* < 0.0001, main effect of diet, *F*_1, 21_ = 0.4240, *P* = 0.522, *N* = 23). **f** Body weight of aged males was significantly changed over time (RM ANOVA, main effect of time, *F*_5, 100_ = 67.034, *P* < 0.0001, *N* = 22). Data represented as mean ± SEM. Repeated measures ANOVA followed by Sidak correction for multiple comparisons. *P < 0.05, **P < 0.01, ***P < 0.001

As such, this study sought to examine two overarching hypotheses on the impact of dietary fat and soluble fiber on gut microbiota. First, we examined the impact of transitioning young and aged male and female mice from a chow diet to a refined low soluble fiber diet on gut microbiota community dynamics. It is important to note that despite the significant differences in dietary fiber composition and content, the refined low-fat/low soluble fiber diet (rLFD) and chow diet are comparable based on proportion of dietary fat and caloric density (Fig. 1a). Second, to examine the extent to which dietary fat alters gut microbiota composition, a subset of animals were switched from a 12% fat refined diet (rLFD) to a 45% fat, low soluble fiber diet (rHFD) for 4 weeks. As the refined diets are formulated using the same compositionally defined ingredients, the rLFD is a more appropriate control diet for these experiments than the chow diet. We used a serial sampling strategy coupled with 16S rRNA marker gene sequencing to assess longitudinal effects of dietary fiber and fat on gut microbiota community structure and composition. Specifically, we assess the impact of transitioning animals from chow to refined diets and the impact of consuming rLFD or rHFD on body weight and gut microbiota. Together, these studies provide insight on critical interactions between diet, sex, and age on the gut microbiota and whole body metabolism.

## Results

### Refined diet promotes weight changes in a sex- and age-specific manner

To examine whether reduction of soluble fiber is associated with body weight alterations in a sex- and age-specific manner, body weight was tracked in young adult and 1-year aged male and female mice across multiple dietary transitions (Fig. 1a). All anima ls were maintained on a chow diet (LabDiet5001) until the initiation of the experiment. Young adult animals consumed the chow diet for 17 weeks, and 1-year aged animals consumed the chow diet for 60 weeks. As the chow diet is formulated from unrefined ingredients that contain on average 12% fat and 15% dietary fiber in the form of soluble and insoluble plant polysaccharides (range of total dietary fiber between 15 and 25% with 15–20% insoluble and 2–5% soluble fiber) [7], we included a 1-week transition period wherein all mice were fed a compositionally defined and refined low-fat/low soluble fiber diet (rLFD, Research Diets AIN76A) that contains 12% fat and 5% insoluble fiber in the form of cellulose. Despite the significant differences in dietary fiber composition and content, the rLFD and chow diet are considered to be comparable based on macronutrient profiles and caloric density (Fig. 1b) [25]. Following the 1-week transition period, half of the animals remained on the rLFD while the remaining half was transitioned to a commonly used refined high-fat/low soluble fiber diet (rHFD, Research Diets D12451) that contains 45% fat and 5% fiber in the form of insoluble cellulose (Fig. 1a). Animals were maintained on rLFD or rHFD for 4 weeks. As the rLFD and rHFD are formulated using the same compositionally defined ingredients, the rLFD is a more appropriate control diet for these experiments than chow.

A comparison of weekly body weights across these defined periods of dietary transitions revealed significant age- and sex-specific effects. Comparison between young adult females revealed a main effect of time, no main effect of diet, and a significant interaction between time and diet (RM ANOVA, main effect of time, *F*_5, 110_ = 15.39, *P* < 0.000, main effect of diet, *F*_1, 22_ = 2.920, *P* = 0.1016, time × diet interaction, *F*_5, 110_ = 2.782, *P* = 0.0210, *N* = 24). Post hoc analysis revealed that young rHFD females showed a higher percentage of body weight gain at week 5 than young rLFD females, but this effect disappeared by week 6 (Tukey with correction for multiple comparisons; 5 weeks, *t*_20_ = 2.278, *P* < 0.05) (Fig. 1c). Body weight comparison in 1-year aged females revealed a main effect of time, a main effect of diet, and a significant time × diet interaction (RM ANOVA, main effect of diet, *F*_1, 18_ = 6.800, *P* = 0.0178, *N* = 20), and their interaction (RM ANOVA, time × diet, *F*_5, 90_ = 12.02, *P* = < 0.0001, *N* = 20). Post hoc analysis revealed aged females fed rHFD weighed more at 2 (*t*_108_ = 3.499, *P* = 0.0041), 3 (*t*_108_ = 3.748, *P* = 0.0017), and 4 (*t*_108_ = 4.781, *P* < 0.0001) weeks compared with rLFD-fed 1-year aged females. These age-specific effects in females may suggest that aged females are more sensitive to lasting high-fat-diet-induced weight gain (Fig. 1d). Males consuming rLFD or rHFD increased body weight across time in a manner that was independent of diet or age (young adult males, main effect of time, *F*_5, 105_ = 88.146, *P* < 0.0001, main effect of diet, *F*_1, 21_ = 0.4240, *P* = 0.522, *N* = 23; 1-year aged adult males, main effect of time, *F*_5, 100_ = 67.034, *P* < 0.0001, *N* = 22; RM ANOVA). These results show that altering sources of fiber, fat, or a combination of both in compositionally defined diets promote weight gain in a sex- and age-specific manner.

### Female-specific changes to fecal microbiota are independent of dietary fat

**Fig. 2:**
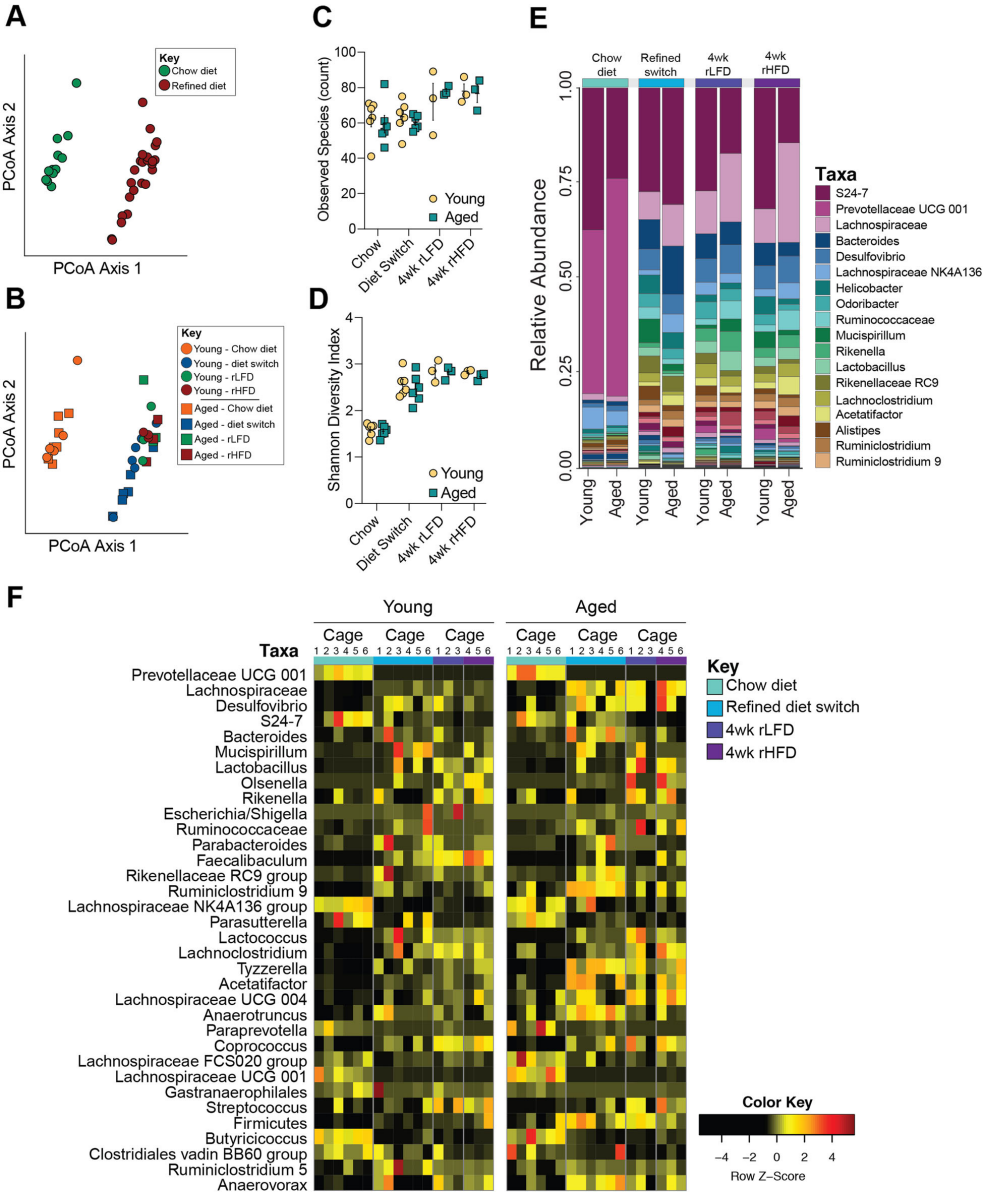
Lack of soluble fiber, not fat, significantly alters gut microbiota composition in young adult and 1-year aged female mice. **a** Principal coordinates analysis comparing fecal microbiota community structure between females consuming chow and refined diet, demonstrating the significant effect of chow and refined diet on community structure (PERMANOVA, *F* = 26.284, *r*^2^ = 0.614, *P* < 0.0001), accounting for 67.4% of variance. No difference in community structure between rLFD and rHFD females was observed (PERMANOVA, *F* = 1.75, *r*^2^ = 0.074, *P =* 0.132). **b** Additional principal coordinates analysis comparing fecal microbiota community structure between young adult and aged females consuming chow, rLFD, and rHFD, demonstrating significant interactions of age, rLFD, and rHFD on community structure (PERMANOVA, *F* = 10.122, *r*^2^ = 0.614, *P* < 0.001). **c**, **d** Comparison of community diversity in young adult and aged females consuming chow, rLFD, and rHFD. **c** The alpha diversity measure, observed species, plotted against sampling time point demonstrating no impact of diet transition and consumption of refined diets on the number of unique taxa (Kruskal-Wallis, *H* = 9.45, *P* = 0.22). **d** The alpha diversity measure, Shannon diversity index, plotted against sampling time point shows significant differences in community richness and evenness in young adult and aged females (Kruskal-Wallis, *H* = 26.647, *P* = 0.00038). **e** Stacked barplot showing the average relative abundance of taxa in chow, rLFD, and rHFD young adult and aged females, characterized by a rapid and lasting loss of taxa within the Bacteroidetes phylum and concomitant bloom of taxa within the Firmicutes and Proteobacteria phyla. Taxa key is truncated to display the 18 most abundant taxa. **f** Heatmap depicting 34 significantly different taxa by age and diet in females identified by linear discriminant analysis (FDR < 0.05). Columns represent taxa within each cage. *N* = 3 female cages/age/diet sampling time point. Data represented as individual data points average per cage ± SEM

Alterations to the gut microbiota have been proposed as a key mechanism contributing to high-fat-diet-induced metabolic dysfunction. These hypotheses stem from studies that compare the gut microbiota of rodents fed a high-fat/low soluble fiber diet to those fed a chow diet [2, 9, 10, 13]. To determine whether the transition from a chow diet that contains soluble fiber to diets that lack soluble fiber and are supplemented with varying proportions of dietary fat influences fecal microbiota, fecal pellets were collected from young adult and 1-year aged female mice consuming chow diet, 1 week following transition to the rLFD and following 4 weeks of consuming rLFD or rHFD. To examine whether microbiota community structure is altered across dietary transitions, beta and alpha diversity measures were calculated and compared as a function of time consuming rLFD and rHFD diets. Bray-Curtis divergence matrices were calculated to assess the distances between young adult and aged females consuming rLFD and rHFD, and then visualized using PCoA (Fig. 2a, b). Permutational multivariate analysis of variance revealed significant effect between chow and refined diets (PERMANOVA, *F* = 26.284, *r*^2^ = 0.614, *P* < 0.0001), indicating that 1 week of consuming a refined diet produced significant community restructuring relative to chow diet females (Fig. 2a). Transitioning young adult and 1-year aged females from rLFD to rHFD did not further change microbiota community structure (PERMANOVA, *F* = 1.75, *r*^2^ = 0.074, *P =* 0.132), indicating that switching female mice from a diet with sufficient levels of soluble fiber (chow) to a diet lacking soluble fiber eclipses the influence of dietary fat on fecal microbiota structure (Fig. 2b). Microbial diversity was significantly altered in some (Shannon diversity index) but not the other (observed species counts) indices of alpha diversity in young adult and aged females (observed species counts, Kruskal-Wallis, *H* = 9.45, *P* = 0.22; Shannon diversity index, Kruskal-Wallis, *H* = 26.647, *P* = 0.00038) (Fig. 2c, d). The significant changes in the Shannon diversity index were driven by the switch from chow to rLFD, and no additional differences were detected between rLFD and rHFD animals.

Consistently, comparison of fecal gut microbiota community between chow and refined diets in young adult and 1-year aged females revealed significant changes in community composition (Fig. 2e). Thus, to determine whether dietary switches impact fecal microbiota composition in young adult and aged female mice, we applied the linear discriminant analysis effect size (LEfSe) method for differential abundance analysis and used a false discovery rate (FDR) cut-off of *q* < 0.05. LEfSe identified 34 taxa that were affected by the switch from chow to refined diets in young adult and aged females (Fig. 2f). Comparison of fecal samples from females consuming a chow diet and 1 week following post-refined diet switch revealed significant decrease of dominant taxa within the phylum Bacteroidetes, including Bacteroidales, Prevotellaceae, and S24-7, and taxa within the phylum Firmicutes and order Clostridia, including *Butyricicoccus* (FDR < 0.05) (Fig. 2f, Additional file 2: Table S1). Conversely, the transition from the chow diet to rLFD increased abundance of taxa within the phylum Proteobacteria, including *Desulfvibrio* and *Escherichia/Shigella*, and taxa within the phylum Firmicutes and order Clostridia, including *Streptococcus*, *Mucispirillum*, *Coprococcus*, *Olsenella*, *Ruminoclostridium*, *Faecalibaculum*, *Acetatifactor*, *Rikenella*, and *Lactobacillus* (FDR < 0.05). Similarly, switching females from rLFD to rHFD either maintained taxa that were altered during the initial transition from chow to rLFD. Switching females from rLFD to rHFD maintained differences that occurred during the transition from chow to rLFD, suggesting that alterations to gut microbiota composition in young adult and aged females are driven by the lack of soluble fiber in the refined diets rather than proportion of fat (Fig. 2f, Additional file 2: Table S1). In a subsequent analysis, we removed chow diet samples and observed no differences in microbiota structure, diversity, or composition between rLFD and rHFD young adult and aged females (all FDR values > 0.05) (Fig. 2f, Additional file 2: Table S1).

Based on our observation that rHFD aged females significantly gain more body weight than aged rLFD females, we next examined whether resistance to weight gain in young adult females and susceptibility to weight gain in aged females is associated with changes to gut microbiota composition. Differential abundance analysis revealed that aged rHFD females had increased abundance of *Bifidobacterium* relative to young adult rHFD females (FDR *q* = 0.0167). No other differences in gut microbiota composition were detected in females.

### Male-specific changes to fecal microbiota are independent of dietary fat

**Fig. 3:**
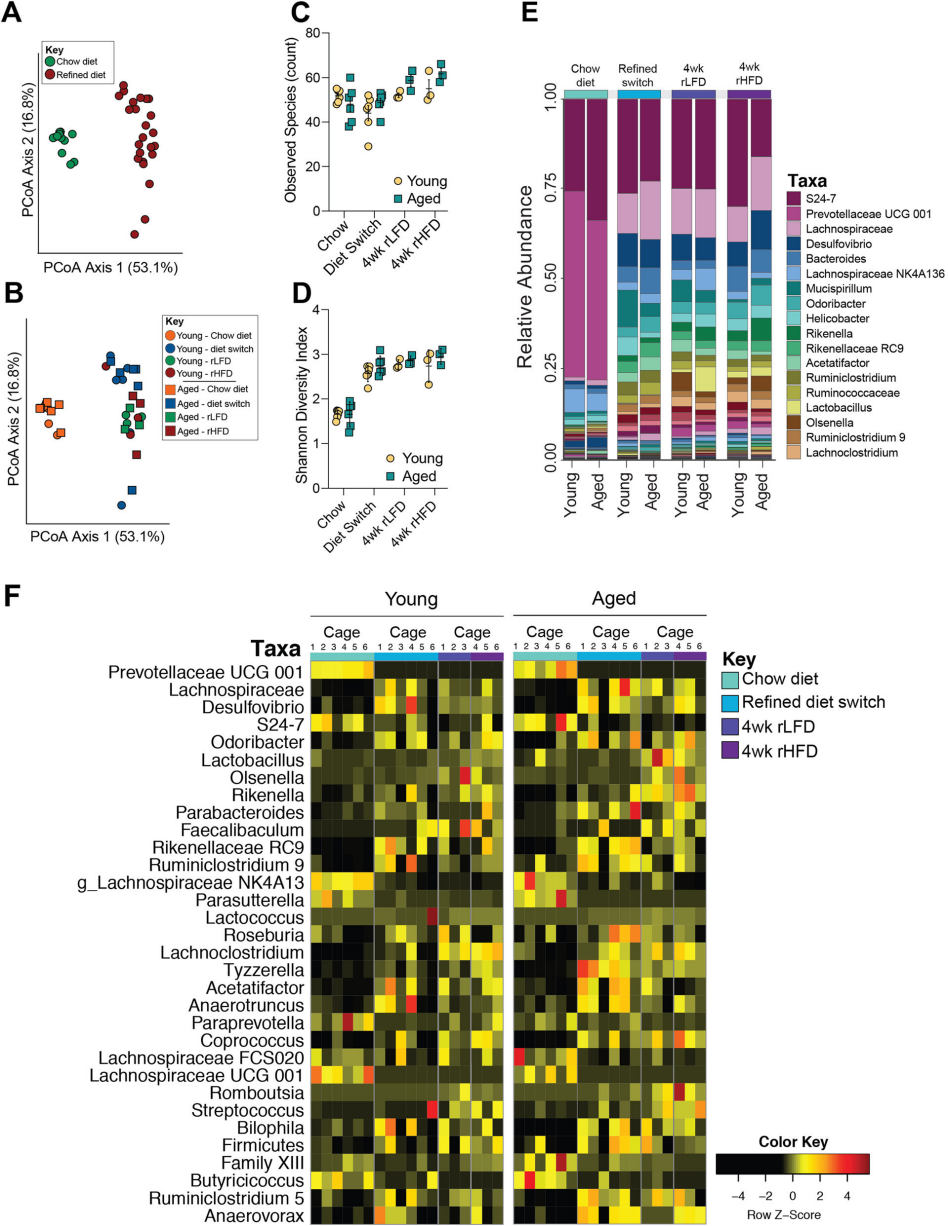
Lack of soluble fiber, not fat, significantly alters gut microbiota composition in young adult and 1-year aged male mice. **a** Principal coordinates analysis comparing fecal microbiota community structure between males consuming chow and refined diet, demonstrating the significant effect of chow and refined diet on community structure (PERMANOVA, *F* = 26.577, *r*^2^ = 0.617, *P* < 0.0001), accounting for 69.9% of variance. No difference in community structure between rLFD and rHFD males was observed (PERMANOVA, *F* = 1.264, *r*^2^ = 0.054, *P =* 0.261). **b** Additional principal coordinates analysis comparing fecal microbiota community structure between young adult and aged males consuming chow, rLFD, and rHFD, demonstrating significant interactions of age, rLFD, and rHFD on community structure (PERMANOVA, *F* = 8.84, *r*^2^ = 0.688, *P* < 0.001). **c**, **d** Comparison of community diversity in young adult and aged males consuming chow, rLFD, and rHFD. **c** The alpha diversity measure, observed species, plotted against sampling time point demonstrating significant differences in the number of unique taxa following dietary transition and consumption of refined diets (Kruskal-Wallis, *H* = 17.32, *P* = 0.015). **d** The alpha diversity measure, Shannon diversity index, plotted against sampling time point shows significant differences in community richness and evenness in young adult and aged males (Kruskal-Wallis, *H* = 26.943, *P* = 0.00034). **e** Stacked barplot showing the average relative abundance of taxa in chow, rLFD and rHFD young adult and aged males, characterized by a rapid and lasting loss of taxa within the Bacteroidetes phylum and concurrent bloom of taxa within the Firmicutes and Proteobacteria phyla. Taxa key is truncated at the 18 most abundant taxa. **f** Heatmap depicting 32 significantly different taxa by age and diet in females identified by linear discriminant analysis (FDR < 0.05). Columns represent taxa within each cage. Data represented as individual data points averaged per cage ± SEM

Similar to the patterns observed in females, community structure of fecal microbiota is significantly different between young adult and aged males following the switch from chow to rLFD (PERMANOVA, *F* = 26.577, *r*^2^ = 0.617, *P* < 0.0001). No difference in community structure between rLFD and rHFD males was observed (PERMANOVA, *F* = 1.264, *r*^2^ = 0.054, *P =* 0.261) (Fig. 3a, b). Microbial diversity was significantly different between young adult and aged males (observed species count, Kruskal-Wallis, *H* = 17.32, *P* = 0.015; Shannon diversity index, Kruskal-Wallis, *H* = 26.943, *P* = 0.00034) (Fig. 3c, d). For both indices of alpha diversity, the significant changes were driven by the switch from chow to rLFD, and no additional differences were detected between rLFD and rHFD animals.

Consistent with alterations to community structure and diversity, the fecal microbiota between chow and refined diets revealed significant remodeling (Fig. 2e). Differential abundance analysis by LEfSe identified 32 taxa that differed between chow and refined diet-fed males. The transition from chow to rLFD resulted in a significant decrease in taxa within the Bacteroidetes phylum, including Prevotellaceae and Lachnospiraceae, and taxa within the Firmicutes phylum, including *Butyricicoccus* (FDR < 0.05) (Fig. 3e, Additional file 3: Table S2). Concurrently, we observed a significant increase in the abundance of taxa within the Proteobacteria phylum, including Desulfvibrio and Parasutterella, and taxa within the phylum Firmicutes, including *Roseburia*, *Coprococcus*, *Bilophila*, *Olsenella*, *Faecalibaculum*, *Acetatifactor*, *Rikenella*, and *Lactobacillus* (FDR < 0.05). Similar to females, switching males from rLFD to rHFD maintained differences in taxa that were altered during the switch from chow to rLFD. In a subsequent analysis, we removed chow diet samples and observed no differences in microbiota structure, diversity, or composition between rLFD and rHFD young adult and aged males (all FDR values > 0.05) (Fig. 2e, Additional file 3: Table S2). No other differences in gut microbiota composition were detected in males. Taken together, these results demonstrate that removal of soluble fiber is an important driver of gut microbiota alterations, while varying proportions of dietary fat may exacerbate these changes.

### Sex-specific effects on gut microbiota composition following the transition to refined diets

Given the significant interaction between sex, age, and diet on gut microbiota community structure and composition, we next examined whether the gut microbiota composition between males and females differed following dietary transitions. Additional analysis revealed that males and females shared 27 taxa that were differentially abundant following the transition to refined diet (FDR < 0.05) (Additional file 1: Figure S1A). Seven taxa were differentially abundant in young adult and aged females, while five taxa were differentially abundant in young adult and aged males (FDR < 0.05) (Additional file 1: Figure S2B-G). These results contribute to a growing literature demonstrating sex-specific diet effects on gut microbiota community and composition and highlight important sex and age interactions on diet, gut microbiota, and body weight.

## Discussion

Dietary intervention studies largely revolve around altering fat content, as diet-induced obesity is comorbid with a variety of serious health conditions in humans, including metabolic disorders, type 2 diabetes, heart disease, liver disease, mood disorders, and cancer [26–28]. However, rodent and nonhuman primate studies often overlook other components of diet that could contribute to disease risk, most notably fiber [29]. In the “Westernized diet” that is often the target of these studies, little consideration has been given to the amount of fiber and whether or not it is soluble. Studies that do account for differences in fiber source in diet formulations demonstrate that high-fat-diet-induced metabolic syndrome and obesity in adult male mice are driven by the lack of soluble fiber in refined diet formulations [8, 29, 30]. Furthermore, other potentially important dietary components have not been considered, therefore limiting the capacity to isolate the effects of the dietary challenge specifically to the fat content. This is a major bottleneck in understanding how diet is involved in the pathophysiology of metabolic and related disorders. The literature on age- and sex-specific effects of high-fat diet consumption on metabolism and gut microbiota is subsequently based on studies comparing mice fed a high-fat/low soluble fiber diet to those fed a chow diet [31–33]. Thus, we examined the age- and sex-specific effect of a refined high-fat/low fiber diet (rHFD) on body weight and gut microbiota composition relative to mice fed a refined low-fat diet (rLFD) that is nutritionally and compositionally matched to the rHFD.

To determine the contribution of soluble fiber and dietary fat on whole body metabolism, body weight measurements were collected from young adult and 1-year aged male and female mice while consuming chow and refined diets. After 4 weeks, young adult female mice showed resistance to weight gain to rHFD, consistent with previous reports [34–36]. Conversely, aged females fed rHFD showed rapid body weight gain relative to rLFD-fed aged females. Age-specific vulnerability to diet-induced body weight gain in females may be related to aging-related changes to estrogens. Circulating estrogens have been shown to be protective against diet-induced obesity by promoting glucose tolerance and insulin resistance, as well as engaging in the direct inhibition of proinflammatory cytokines that are stimulated by consumption of a high-fat diet [35–37]. Reduction in circulating estrogens through ovariectomy dramatically increases risk for high-fat-diet-induced obesity and metabolic disorders in mice and post-menopausal women, providing a possible explanation for rHFD-induced weight gain in aged females [20].

Conversely, young adult and 1-year aged males showed a significant gain in body weight that was independent of refined diet formulation. The significant increase in body weight in young adult and aged males fed either rLFD or rHFD may suggest that other components of the refined diet contribute to body weight gain that is independent of dietary fat [8, 17, 29]. Consistent with our observations, increased body weight and adiposity, loss of intestinal mass, and decreased availability of short chain fatty acids have been previously reported in young adult male mice fed the rLFD relative to chow diet-fed males [8, 29]. Taken together, these results suggest that a lack of soluble fiber and varying proportions of dietary fat exhibit significant effects on body weight in an age- and sex-specific manner, and further highlight the importance of an appropriate refined diet control for diet-induced obesity studies assessing metabolic outcomes.

High-fat-diet-induced weight gain and metabolic dysfunction have been mechanistically linked to altered gut microbiota composition and functional capacity to harvest energy; however, these conclusions are drawn from studies comparing the gut microbiota of rodents fed a high-fat/low soluble fiber diet to those fed a chow diet [2, 3, 11, 12, 37]. To determine whether switching mice from a chow diet to refined diets influences fecal microbiota, fecal pellets were collected from young adult and aged male and female mice while consuming chow diet, 1 week following the transition to the rLFD and following 4 weeks of consuming rLFD or rHFD. The transition from the chow diet to rLFD resulted in changes to microbiota community structure and composition in all groups, regardless of sex and age. This dietary transition was characterized by a loss of taxa within the phylum Bacteroidetes and a concomitant bloom of Clostridia and Proteobacteria in a sex- and age-specific manner. Specifically, the bloom of *Escherichia/Shigella* in females, but not males, is an interesting observation for future investigation given the interaction between nutritional environment of the gut, bloom of Proteobacteria, and systemic inflammation [38]. Similar changes to gut microbiota have been previously reported in mice consuming a rHFD, suggesting that the bloom of Clostridia and Proteobacteria may be attributed to loss of soluble fiber rather than the high proportion of dietary fat [37]. Further, dramatic changes in gut microbiota composition within 1 week of switching from a chow diet to the rLFD highlight the exquisite sensitivity of the gastrointestinal ecosystem to shifts in nutrient availability and composition. Given the high diet-to-diet and batch-to-batch variability in the total fiber levels and concentrations of each fiber type, it is difficult to generalize chow diet formulation across studies and further raises questions as to the use of chow diet as an appropriate control diet [39, 40]. The lack of differences between rLFD- and rHFD-fed mice may indicate that gut microbiota structure and composition can be dissociated from body weight and systemic inflammation [17, 29, 30]. Moreover, given the well-established influence of host genetics on microbiota and metabolism, additional studies are needed to determine the sex- and strain-specific impact of rHFD and rLFD on gut microbiota composition and phenotypic outcomes [41]. Further, these results suggest that the absence of soluble fiber in both refined diets exerts a more dominant effect than the varying proportions of fat between these diets.

## Conclusion

Collectively, our results highlight that the choice of control diet for diet-induced metabolic disease experiments may influence interpretations related to the role of gut microbiota in experimental readouts. These data also have broad implications for rodent studies that draw comparisons between refined high-fat/low soluble fiber diets and chow diets to examine dietary fat effects on metabolic, immune, behavioral, and neurobiological outcomes. Moving forward, it will be of great interest to examine the mechanisms underlying the sex differences of soluble fiber in whole body metabolism and determine whether compositionally defined combinations of fiber and fat influence metabolism, immunity, and neural circuits that control feeding behavior and energy homeostasis.

## Methods

### Animals

Male and female mice used in this study were derived from an in-house C57Bl/6:129 mixed strain. Mice were housed in a 12-h light-dark cycle. Food (LabDiet 5001) and water were provided ad libitum. Mice were group housed following weaning at postnatal day 28. For the young adult cohort, the average age at the start of the study was 17 weeks old. For the aged cohort, the average age was 60 weeks old.

### Experimental design

Figure 1a provides an overview of the experimental design. All mice were maintained on chow diet (LabDiet 5001) and fresh fecal pellets were collected prior to refined diet transition. On average, the chow diet supplied energy as 13.4% fat, 28% protein, 57.9% carbohydrates, and 15% dietary fiber (range of total dietary fiber between 15 and 25% with 15–20% insoluble and 2–5% soluble fiber). Two refined diets were used during the experiment: refined low-fat/low soluble fiber diet (rLFD, Research Diets AIN76A) supplying energy as 12% fat, 21% protein, and 67% carbohydrates; and refined high-fat/low soluble fiber diet (rHFD, Research Diets D12451) supplying energy as 45% fat, 20% protein, and 35% carbohydrates. First, all mice were placed on the rLFD for 1 week to acclimate them to a refined diet. Following acclimation, mice were randomized to either remain on rLFD or switch to rHFD. At that point, a total of 12 young adult males, 12 young adult females, 11 aged males, and 10 aged females remained on rLFD, and a total of 12 young adult males, 12 young adult females, 12 aged males, and 11 aged females were placed on rHFD. Diets were available for ad libitum consumption. Body weight was recorded once per week. Fecal pellets were collected following the 1-week acclimation period and 4 weeks post-diet transition. While individual fecal pellets were collected, mice remained group housed for the entire course of the experiment. As group housing condition has been shown to account for a significant portion of variance in fecal microbiota community composition, all analysis was conducted at the level of the cage (*N* = 3 cages per sex/diet, total *N* = 92 mice).

### DNA extraction

Genomic DNA from fecal samples were isolated using the Stratec PSP Spin Stool DNA Plus kit using the difficult to lyse bacteria protocol from the manufacturer (STRATEC Molecular GmbH, Berlin, Germany). Each sample DNA was eluted into 100 μL of elution buffer provided by the Stratec PSP Spin Stool DNA Plus kit.

### Illumina MiSeq 16S rRNA marker gene sequence data processing and analysis

The V4 region of the bacterial 16S rRNA gene was amplified using a dual-index paired-end sequencing strategy for the Illumina platform as previously described [42]. Sequencing was performed on a MiSeq instrument (Illumina, San Diego, CA) using 2 × 250 base paired-end chemistry at the University of Maryland School of Medicine Institute for Genome Sciences. The sequencing run yielded a total of 25,260,257 read counts with an average of 33,292 read counts per sample. The sequences were demultiplexed using the dual-barcode strategy, a mapping file linking barcode to samples and split_libraries.py, a QIIME-dependent script [43]. The resulting forward and reverse fastq files were split by sample using the QIIME-dependent script split_sequence_file_on_sample_ids.py, and primer sequences were removed using TagCleaner (version 0.16) [44]. Further processing followed the DADA2 workflow for Big Data and DADA2 (v.1.5.2) (https://benjjneb.github.io/dada2/bigdata.html) [45]. Data filtering was set to include features where 20% of its values contain a minimum of 4 counts. In addition, features that exhibit low variance across treatment conditions are unlikely to be associated with treatment conditions, and therefore, variance was measured by inter-quartile range and removed at 10%. Data was normalized by cumulative sum scaling, and differential abundance analysis was conducted using linear discriminant analysis effect size with an FDR cut-off at *q* < 0.05 [46]. For quality control purposes, water and processed blank samples were sequenced and analyzed through the bioinformatics pipeline. Taxa identified as cyanobacteria or “unclassified” to the phylum level were removed. The sequencing data has been deposited in the Sequence Read Archive (SRA) of the National Center for Biotechnology Information (NCBI) (Bioproject: PRJNA6083825) to be released upon publication.

### Statistical analysis

Data are represented as individual data points or individual points averaged per cage ± SEM. Body weight data were analyzed by repeated measures analysis of variance, and post hoc analysis was conducted using Sidak correction for multiple comparisons. Given the nonparametric nature of microbiota data, indices of alpha diversity data were analyzed using the Kruskal-Wallis test. Permutational multivariate analysis of variance was used to analyze effects of diet, sex, and age. 16S rRNA marker gene sequencing raw count data was filtered and was set to include features where 20% of its values contain a minimum of 4 counts. In addition, features that exhibit low variance across treatment conditions are unlikely to be associated with treatment conditions, and therefore, variance was measured by inter-quartile range and removed at 10%. Data was normalized by cumulative sum scaling, and differential abundance analysis was conducted using linear discriminant analysis effect size with an FDR cut-off at *q* < 0.05. Analysis was conducted in the R environment and Bioconductor using the metagenomeSeq package, heatmaps were visualized using the heatmap.2 function within the gplots package, and bar plots were visualized using GraphPad [47–50].

## Supplementary information

**Additional file 1: Figure S1.** Sex-specific alterations of refined diet on gut microbiota composition. (A) Venn diagram depicting microbiota significantly altered by diet in young adult and aged males and females (27), female-specific taxa altered by diet (7) and male-specific taxa altered by diet (5) identified by linear discriminant analysis (FDR < 0.05, N = 3 cages/sex/age/diet sampling timepoint). (B-G) Taxa abundance plotted against sampling time point showing sex-specific differences identified by linear discriminant analysis (FDR = 0.05). Data represented as individual data points averaged per cage ± SEM.

**Additional file 2: Table S1.** Results from linear discriminant analysis of microbiota composition in female mice.

**Additional file 3: Table S2.** Results from linear discriminant analysis of microbiota composition in male mice.

**Additional file 4: Table S3.** Metadata and taxonomic classifications.

## Data Availability

No custom code or software was used for the analysis discussed in this manuscript. Metadata and taxonomic classifications are provided in Additional file 4: Table S3.

## Abbreviations

ANOVA: Analysis of variance
FDR: False discovery rate
PCoA: Principle coordinates analysis
PERMANOVA: Permutational analysis of variance
rHFD: Refined high-fat diet
rLFD: Refined low-fat diet
RM ANOVA: Repeated measures analysis of variance
rRNA: Ribosomal RNA
SCFA: Short chain fatty acid
