# Supplementary figures and images for: It's the fiber, not the fat: significant effects of dietary challenge on the gut microbiome
Source: Microbiome. 2020 Feb 11;8:15. doi: 10.1186/s40168-020-0791-6 (PMC7014620; doi:10.1186/s40168-020-0791-6)

**Figure S1**


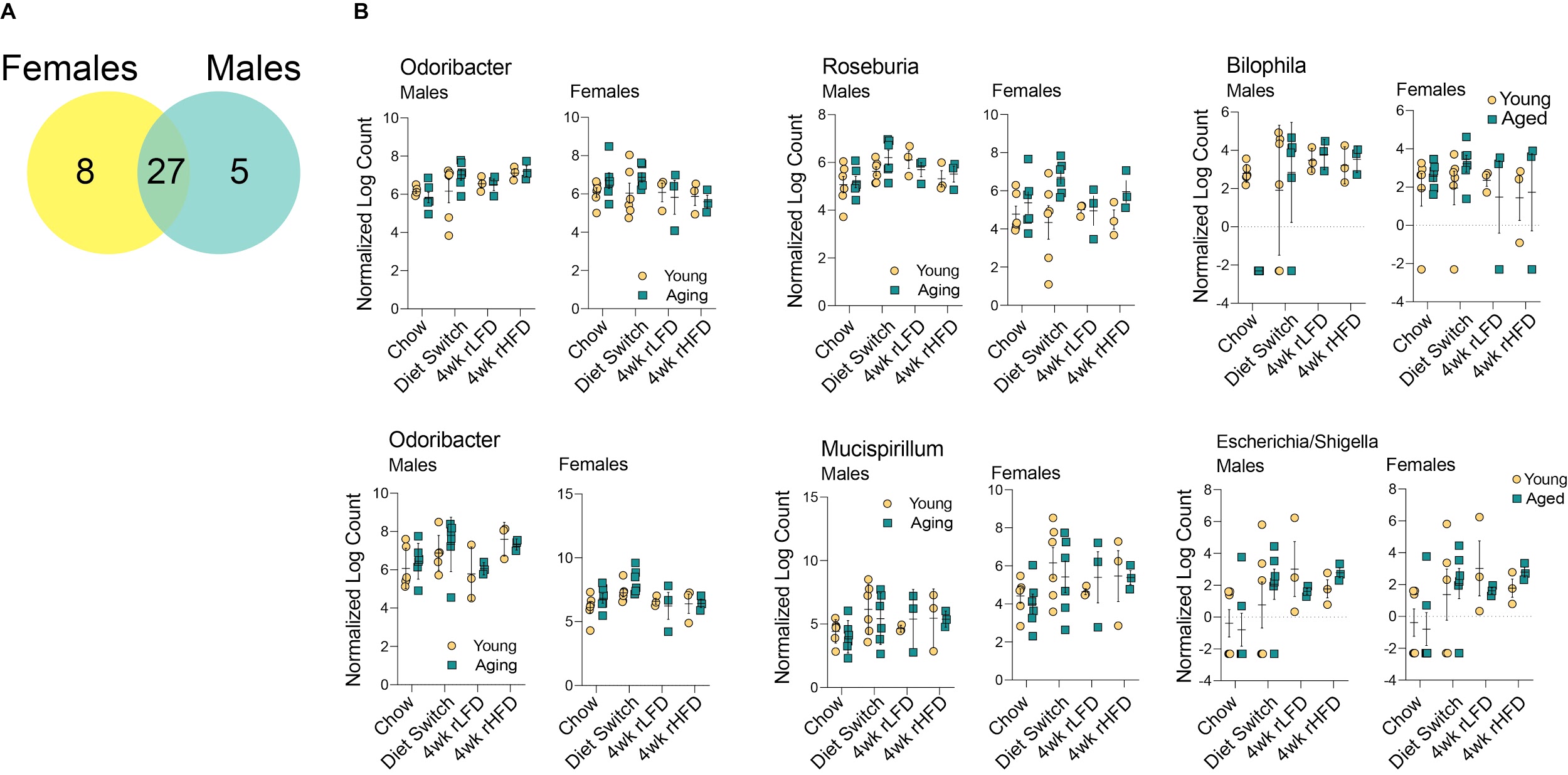

Supplement: Supplementary file 2 — Additional file 1: Figure S1. Sex-specific alterations of refined diet on gut microbiota composition. (A) Venn diagram depicting microbiota significantly altered by diet in young adult and aged males and females (27), female-specific taxa altered by diet (7) and male-specific taxa altered by diet (5) identified by linear discriminant analysis (FDR < 0.05, N = 3 cages/sex/age/diet sampling timepoint). (B-G) Taxa abundance plotted against sampling time point showing sex-specific differences identified by linear discriminant analysis (FDR = 0.05). Data represented as individual data points averaged per cage ± SEM. [file 40168_2020_791_MOESM1_ESM.docx]
